# Private Face Image Generation Method Based on Deidentification in Low Light

**DOI:** 10.1155/2022/5818180

**Published:** 2022-03-17

**Authors:** Beibei Dong, Zhenyu Wang, Zhihao Gu, Jingjing Yang

**Affiliations:** ^1^School of Information Science and Engineering, Hebei North University, Zhangjiakou 075000, China; ^2^Sifang College, Shijiazhuang Tiedao University, Shijiazhuang 051132, China

## Abstract

The existing face image recognition algorithm can accurately identify underexposed facial images, but the abuse of face image recognition technology can associate face features with personally identifiable information, resulting in privacy disclosure of the users. The paper puts forward a method for private face image generation based on deidentification under low light. First of all, the light enhancement and attenuation networks are pretrained using the training set, and low-light face images in the test set are input into the light enhancement network for photo enhancement. Then the facial area is captured by the face interception network, and corresponding latent code will be created through the latent code generation network and feature disentanglement will be done. Tiny noise will be added to the latent code by the face generation network to create deidentified face images which will be input in a light attenuation network to generate private facial images in a low-lighting style. At last, experiments show that, compared with other state-of-the-art algorithms, this method is more successful in generating low-light private face images with the most similar structure to original photos. It protects users' privacy effectively by reducing the accuracy of the face recognition network, while also ensuring the practicability of the images.

## 1. Introduction

**Figure 1:**
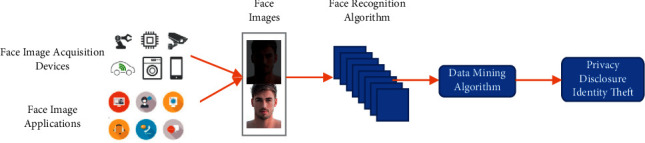
Schematic diagram of privacy leakage of users.

At present, face image recognition technology, based on deep learning technology, has become one of the first choices for identifying and verifying individual identity due to its convenience, efficiency, and maturity [1], and it has been widely applied in the Internet of Things (IoT) and cloud computing [2, 3]. In addition, in the fields of target detection [4], social media data mining [5], and autonomous driving [6, 7], face images are constantly being collected. Face images, however, representing individual characteristics, are of uniqueness and invariability. If they are posted by users or collected passively without any protection of face characters, those characters will inevitably be illegally collected and analyzed [8], thereby resulting in serious identity theft and information fraud, for example, the privacy disclosure incident covering more than 50 million users of Facebook [9] and the illegal profit-making issue of Alipay (a mobile payment software) by forging face images [10]. As shown in Figure 1, face image acquisition devices and applications collect a large number of face images under various lights, and the use of face recognition algorithms and data mining algorithms by criminals will lead to user privacy leakage and identity theft. User privacy leak is detrimental to social stability. The leakage of private data has become a major global social problem in the Internet era, which is universal, frequent, and explosive. Enterprises and users are harassed and violated. Leakage of private data often triggers explosive incidents. Once an incident occurs, it will have serious consequences, with high levels of damage, often producing resonance effects, triggering social dissatisfaction and turbulence, and having a wide range of impacts. In terms of time, it may continue for several years, and it is difficult to eliminate the impact in a short time. Therefore, the European Union formally implemented General Data Protection Regulation in May 2018, clarifying the data rights of citizens and the basis of privacy protection. At the same time, frequent privacy leakage events made users averse to face image recognition technology and they refused to enter places installed with face image acquisition equipment. The above incidents have seriously hindered the application and development of artificial intelligence technology and the Internet of Things.

It has been a hotspot to study how to prevent the abuse of face image recognition technology, remove the association between facial features and personal identity information, and avoid the disclosure of user privacy on the premise of ensuring the practicability of face images. Deidentification of the face has become a potential solution to this problem [11]. Although related studies on face deidentification can already mislead face image recognition algorithms in identity recognition, its effectiveness usually relies on sufficient light [12, 13]. In the low-light environment, changes in ambient light and differences in the object's surface material often result in uneven brightness, unclear image texture, and low contrast of local features. All of these problems will bring great challenges to existing face image deidentification methods. However, existing face image recognition algorithms have long been able to accurately identify underexposed face images [14, 15]. After our experiments on existing face deidentification methods in low-light environments, the success rate of generating private face images cannot be guaranteed due to the failed generation or the generated images being too dark. If low-light private face images could not be generated, then users' privacy will not be well protected due to the existence of low-light face recognition algorithms. Therefore, it is crucial to overcome the impact of low light on face image deidentification. The existing deidentification methods have a low success rate in generating low-light-style private face images. We should look for and achieve a new method for low-light private image generation based on face image deidentification. It can eliminate or reduce unfavorable factors caused by low light, allowing generated face images to show more details and features. It can also generate low-light, deidentified face images, which are extremely similar to the brightness and contrast of the original images. Otherwise, the faces will be not natural enough in low-light scenes, and the user experience will be deteriorated.

The paper proposes a face privacy image generation method based on deidentification and under low light. It has a higher privacy protection capacity with fewer processing traces and good visual quality. The main contributions of the paper are as follows:

1. The method in this paper designs the light enhancement network based on the Retinex theory. The low-light face image is enhanced by the light enhancement network and then face deidentification is performed, which overcomes the adverse effects of low light and improves the success rate of generating deidentified private face images under low light.
2. The method in this paper trains the light attenuation network with the opposite training strategy of the light enhancement network to generate low-light style face privacy images. The face privacy images are real and natural under low light, which improve the user's experience.
3. Although the features of the face region of the face privacy images generated by this method are obviously different from the original face images, they still maintain the basic appearance of the original face images. The method in this paper ensures the practicability of generating face privacy images and, at the same time, misleads face image recognition network recognition and protects user privacy.

The structure of the thesis is as follows: The second part introduces relevant studies, the third part explains the method proposed in this paper, the fourth part is about experiments and analysis, and the fifth part summarizes the whole paper.

## 2. Related Work

### 2.1. Generative Adversarial Networks

The classic deidentification method is based on cryptography. However, a large number of computing resources are required, which is not conducive to real-time transmission. In the current popular research, private face image generation methods based on deidentification are divided into the deidentification method based on face disturbance [16], the deidentification method based on face mixing [17], and the deidentification method based on deep learning. Thereinto, face privacy images generated by the deep learning-based deidentification method are of higher image quality with stronger privacy protection capacity, so it has become a hot research topic. The basis of deidentification methods is the generation of virtual faces. The generation of virtual faces is mainly realized by using the GAN (Generative Adversarial Networks) proposed by Goodfellow et al. [18]. GAN is structurally inspired by the two-person zero-sum game in game theory. It sets the two parties participating in the game as a generator and a discriminator. The purpose of the generator is to learn and capture the truth as much as possible, learn the potential distribution of data samples, and generate new data samples. The discriminator is a binary classifier whose purpose is to correctly judge whether the input data comes from real data or the generator. To win the game, these two game participants need to be continuously optimized. Each improves its own generation ability and discriminative ability. This learning optimization process is a Minimax game problem. The purpose is to find a Nash equilibrium between the two so that the generator can estimate the distributed data samples. Various GAN-based derivative models are proposed to improve the structure of the model and further expand the theory and apply it. Arjovsky et al. [19] proposed Wasserstein GAN (WGAN), which solves the problem of gradient disappearance caused by discontinuity of the optimization target. Radford et al. [20] proposed DCGAN (Unsupervised Representation Learning with Deep Convolutional Generative Adversarial Networks), which uses convolutional neural networks for supervised learning and GAN for unsupervised learning to generate images and obtains relatively good results to verify the generated image feature representation expressive ability. GAN can generate images, videos, etc., and has a very wide range of applications. In this paper, GAN is used to generate private images of human faces.

### 2.2. Deidentification Method Based on Deep Learning

Among the method of deidentification methods based on deep learning, Karras et al. [21] proposed PGGAN which makes the generation of high-quality and high-resolution images possible through a progressive approach. PGGAN proposes the concept of layer-by-layer training, but it also increases the complexity of training. Then they proposed the epoch-making StyleGAN [22] on this basis, which untangles latent code through a nonlinear mapping network to control high-level attributes of generated images. Aiming at the “water droplets” in images generated by StyleGAN, Karras et al. redesigned the normalization scheme used in the generator and put forward StyleGAN2 [23], solving the artifact problem of generated images. StyleGAN2 can generate high-quality virtual face images but does not achieve good equivariance. Shen et al. proposed InterFaceGAN [24], analyzed semantic characteristics of latent code, and constructed the theory of facial attribute editing through latent code. Grounded on virtual face generation technology, Wu et al. [12] presented PP-GAN for deidentification. It could generate private images of faces with a Generative Adversarial Network (GAN) to avoid its identification by face image recognition systems. Besides, a new validator and modulator were adopted to ensure the quality of private facial images but only experimented on black and white datasets. Based on Generative Adversarial Network and U-NET, He et al. [25] added tiny perturbation to each face image to make deidentified faces wrongly classified by face recognition network, but the “checkerboard effect” arose in deidentified faces. Yang et al. [13] proposed that principal component analysis of faces should be carried out to reduce data redundancy. Then the principal component of face images would be disturbed by adversarial samples and transformed into face images through PCA inversion. However, the quality of generated deidentified face images still needs to be improved. Proenca put forward UU-Net [26], which used Conditional Generative Adversarial Network to create synthetic face privacy images that retain the original posture, lighting, background information, and facial expressions. Lin et al. [27] proposed FPGAN (face deidentification method with generative adversarial networks for social robots). The pixel loss and content loss functions are designed to retain part of the link between the deidentified image and the original image, and U-Net is improved as a generator and applied to the deidentification of social robots. So far, there has been no research on deidentification aiming at private face image generation in low light yet. The method in this paper can break through the flaw of existing technologies which can only be applied under sufficient light and realize deidentification of face image data under low-light conditions, extending application scenarios of the face image deidentification method based on privacy protection.

## 3. The Proposed Method

### 3.1. Definition of the Problem

Suppose there is a low-light face data set from IoT devices, *X*={(*x*_1_, *y*_1_), (*x*_2_, *y*_2_),…, (*x*_*N*_, *y*_*N*_)}. For any low-light face image *x*_*i*_ and *x*_*i*_ ∈ *R*^*m*×*n*^ , the corresponding identity tag is *y*_*i*_. The algorithm of *Q* generates corresponding underexposed face privacy images. Then for random face image recognition algorithm, there is

(1) $$\begin{array}{c} \min_{t}{\left\|\delta \right\|}_{2},\\ s.\text{t}.\;\log\frac{\mathrm{Pr}\left[f\left(x_{i}^{\prime }\right)=y_{i}\right]}{\mathrm{Pr}\left[f\left(x_{i}\right)=y_{i}\right]}<\in . \end{array}$$

Among them, *δ* represents the change amplitude of low-light face image *x*_*i*_ and low-light private face image *x*_*i*_′. To ensure the high practicability of the private face image *x*_*i*_′, *δ* should be as small as possible. The face should be as real and natural as possible, and the brightness, contrast, and other indicators should be as similar as possible to low-light face images *x*_*i*_. *ϵ* is the index of privacy protection degree. For the random face image recognition algorithm *f*, the probability of recognizing the real identity tag *y*_*i*_, corresponding to low-light face privacy images, should be minimized to realize privacy protection. The smaller the *ϵ* is, the better the privacy protection will be. The purpose of this paper is to generate low-light private face images *x*_*i*_′ on the premise of minimizing *x*_*i*_ and *δ*.

### 3.2. The Framework of the Proposed Method

#### 3.2.1. The Overall Framework of the Proposed Method

**Figure 2:**
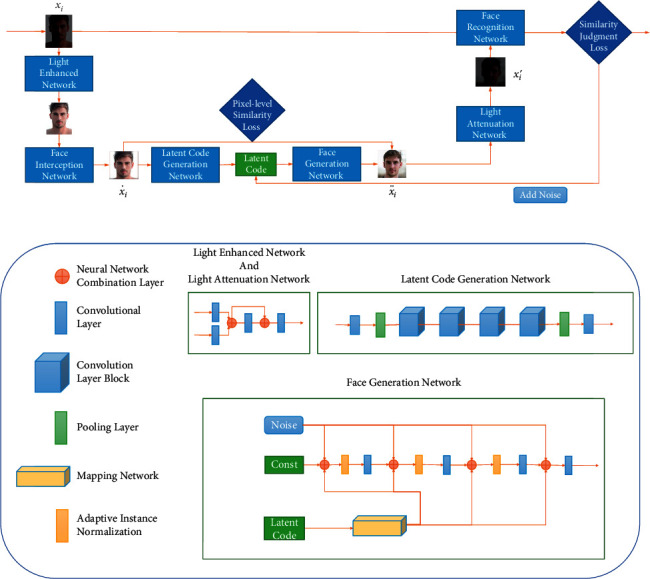
The overall framework of the proposed method.

The overall framework of the low-light private face image generation method based on deidentification is shown in Figure 2. To ensure the high practicability of the generated private face image *x*_*i*_′, the low-light face images are firstly enhanced through a light enhancement network, and the face area is captured through a face cropping network. Then an enhanced face image ${\dot{x}}_{i}$ is created. Then, a private face image ${\dot{x}}_{i}$ is input into the latent code generation net for latent code generation, and the latent code feature is disentangled through the mapping network of the latent code generation net. Then tiny noise is added to the enhanced face image ${\dot{x}}_{i}$ with a synthesis network. Next, Pixel-Level Similarity Loss is adapted to constrain the similarity between the generated face and the enhanced face image ${\dot{x}}_{i}$, to create a deidentified face image ${\ddot{x}}_{i}$ similar to the enhanced face image ${\dot{x}}_{i}$. Deidentified face image ${\ddot{x}}_{i}$ is input in the light attenuation network to generate private face image ${\dot{x}}_{i}$ in low-light style. To ensure that private face image *x*_*i*_′ can successfully mislead face image recognition networks in face identification, it is input in the face image recognition network and Similarity Judgment Loss is set. If the face image recognition network can successfully recognize the face, the noise will be added to the latent code to generate a new deidentified face image ${\ddot{x}}_{i}$. This step will be repeated until the face image recognition network fails in face recognition. Then the private face image *x*_*i*_′ will be output. Among them, the light enhancement and attenuation network are pretrained with paired low-light face images and normal face images. In order to ensure the privacy of the generated face images, the face recognition network adopts a well-trained model with high accuracy.

#### 3.2.2. The Light Enhancement and Attenuation Networks

**Figure 3:**
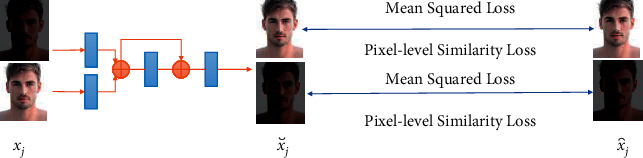
Training schematic diagram of light enhancement and attenuation networks.

The training principle of light enhancement and attenuation networks is shown in Figure 3. Use a specific method to perform low-light processing on the normal-light image to obtain a low-light image paired with it. A low-light face image and a normal-light face image are paired as $\left\{x_{j},{\overset{⌢}{x}}_{j}\right\}$. The low-light facial image is input into the light enhancement network. The face image ${\overset{⌣}{x}}_{j}$ , output by Mean Squared Error, namely, equation (2), is close to its Euclidean distance with normal-light face image. The opposite strategy will be applied when training the light attenuation network. The normal-light face image is adopted as the input of the light attenuation network, and the face image ${\overset{⌣}{x}}_{j}$, output through loss function (2), approaches the Euclidean distance with a low-light face image. Noise is generated randomly. If the similarity between the deidentified face image and the original face image is too high, it is necessary to use noise to interfere with the latent code of the deidentified face. The latent code of the face image is multiplied by random noise to change the face generated by the target latent code. Through multiple trainings, the similarity between the generated face image and the original face image can be reduced.

(2) $$\begin{array}{c} L_{J}=\frac{1}{N}\sum_{i=1}^{N}{\left({\overset{⌢}{x}}_{j}-h_{\theta }\left(x_{j}\right)\right)}^{2}. \end{array}$$

In the loss function (2), *h*_*θ*_ represents the fitting function of light enhancement and attenuation networks. That is, *h*_*θ*_(*x*_*j*_) is the output of the fitting function ${\overset{⌣}{x}}_{j}$, and N represents the size of the training data set. To ensure the training quality of light enhancement and attenuation networks, SSIM (Structural Similarity) indexes [12, 28], shown in loss function (3), are also adopted to drive the output face image ${\overset{⌣}{x}}_{j}$ to be structurally close to the training images ${\overset{⌢}{x}}_{j}$.

(3) $$\begin{array}{c} L_{s}=\frac{1}{2}\left(1-\mathrm{SSIM}\left(x,y\right)\right). \end{array}$$

In the loss function (3), *x*, *y* represent two face images to be compared for structural similarity. The function of the loss functions *L*_*E*_ and *L*_*W*_ is to enable the deep network to achieve the effect of enhancing or attenuating the light of the face image. For the loss function *L*_*E*_ of the light enhancement network and the loss function *L*_*W*_ of the light attenuation network, there are

(4) $$\begin{array}{c} L_{E}=L_{W}\\ =\chi_{1}L_{J}+\chi_{2}L_{s}. \end{array}$$

### 3.3. The Generation of Private Face Images

The classical network model Senet50 is selected as the model of latent code generation network, taking the enhanced face image ${\dot{x}}_{i}$ as the input of Senet50 and connecting the mapping network at the end of Senet50 network to transform the enhanced face images into latent space code. That is, the disentangled feature is latent code. The latent code can be used to control the style of the generated image. The mapping network consists of six full connection layers. Generated latent code is input into face generation network, and Mean Squared Error, namely, equation (5), is used as the loss function of latent code generation network, making the output of face generation network, namely, deidentified face image ${\ddot{x}}_{i}$, approximate to enhanced face images, to drive the latent code generation network to create latent code of enhanced face images in the initial domain.

(5) $$\begin{array}{c} L_{C}=\frac{1}{N}\sum_{i=1}^{N}{\left({\dot{x}}_{i}-{\ddot{x}}_{i}\right)}^{2}. \end{array}$$

The role of the synthesis network is to generate face images. The synthesis network of face generation network adopts the structure of StyleGAN2 and the loss function of logistic with single gradient penalty, as shown in equation (6), where *D* represents a discriminator, *G* stands for a generator, ∇_*T*_real__^2^ serves as the gradient penalty of real samples, and *r*1_gamma_ is the hyperparameter.

(6) $$\begin{array}{c} L_{D}=\log\left(\exp\left(D\left(G\left(z\right)\right)\right)+1\right)+\log\left(\exp\left(-D\left(x\right)\right)+1\right)+r1_{\text{gamma}}*0.5*\sum \nabla_{{\text{T}}_{\text{real}}}^{2},\\ L_{G}=-\log\left(\exp\left(D\left(G\left(z\right)\right)\right)+1\right). \end{array}$$

To ensure that generated private face images can successfully mislead face image recognition networks in face identification, as shown in formula (7), Similarity Judgment Loss is also set to ensure that the generated deidentified face images ${\ddot{x}}_{i}$ can lead to the failure of the face image recognition network.

(7) $$\begin{array}{c} L_{A}=E_{x\prime }\ell_{f}\left({\ddot{x}}_{i},y,y\prime \right). \end{array}$$

Thereinto *ℓ*_*f*_ represents the fitting function of the face image recognition network. When it identifies the deidentity tag of the deidentified face image as the real label, the loss function will return to a higher value. Then the face generation network will add tiny noise to the latent code and repeat the above generation process until *ℓ*_*f*_ identifies the forged identity tag of the deidentified face image. Similarly, to ensure the quality of generated private face images, the synthesis network also adds SSIM (Structural Similarity) index loss function, so the loss function *L*_*F*_ of the face generation network is shown in equation (8), where *χ*_1_, *χ*_2_, *χ*_3_, and *χ*_4_ are hyperparameters.

(8) $$\begin{array}{c} L_{F}=L_{G}+\chi_{1}L_{D}+\chi_{2}L_{s}+\chi_{3}L_{A}+\chi_{4}L_{C}. \end{array}$$

## 4. Experiments and Analysis

### 4.1. Experimental Settings

The hardware configuration used in the experiment is Intel 8700K CPU, 16G DDR4 memory, and 2070Ti graphics card. The implementation of the algorithm uses Python as the programming language and TensorFlow as the deep learning framework.

VGGFACE2 [29] covers a wide range of poses, ages, and races. It is a large-scale face recognition data containing 3.31 million pictures and 9131 IDs. The average number of pictures per ID is 362.6. Now the structure and model parameters of the trained VGG16, Resnet50, and other networks have been open sourced. The experimental data set adopted the public face data set VGGFACE2 from which 300,000 face images were randomly selected. All face images were converted into low-light face images through a new training method of the low-light environment data set [30]. The data set was divided into a training set, validation set, and test set according to the ratio of 98 : 1 : 1. The classical networks VGG16, Resnet50, MobileNet V3, and Senet50 were trained, respectively, to serve as face image recognition networks in the loss function by using transfer learning. All these face image recognition networks adopt triples to construct loss functions, so they all set the threshold of 0.3 to determine whether the input face images belong to the category, as shown in Table 1. To prove the advancement of the method, threshold settings were all equal to or less than the common threshold (0.7–0.9) set by face image recognition networks. Four face image recognition networks all achieved a high recognition rate. Thereinto, True Positive Rate and False Positive Rate are two commonly used indicators in face recognition, and the calculation method is shown in formula (9). TP is correctly classified by the classifier as a positive example; TN is correctly classified by the classifier as a negative example, FP is wrongly classified by the classifier and it should be a negative example, and FN is wrongly classified by the classifier as it is a positive example. Therefore, TPR represents the rate of correctly judged positive among all positive samples. FPR represents the rate of false positives among all negative samples.


(9) $$\begin{array}{c} \left\{\begin{array}{c} \text{TPR}=\frac{\text{TP}}{\text{TP}+\text{FN}},\\ \text{FPR}=\frac{\text{FP}}{\text{FP}+\text{TN}}. \end{array}\right. \end{array}$$

**Figure 4:**
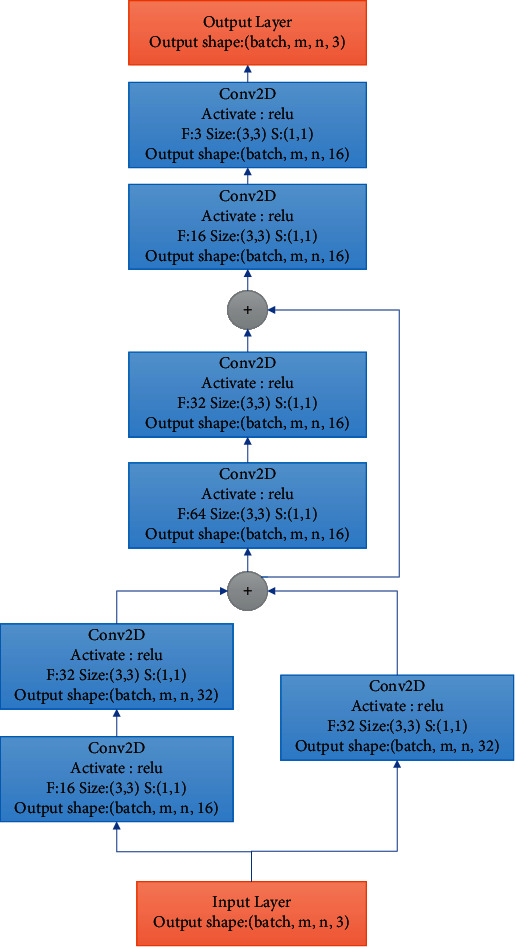
The structure of light enhancement and attenuation networks.

In recent years, deep learning has been widely used in research related to light enhancement such as dehazing and harsh environments [31–33]. Inspired by this, the method in this paper designs a deep neural network for light enhancement and attenuation of face images. The structure of light enhancement and attenuation networks is shown in Figure 4. About the Retinex theory [34], the network was designed as a cascade structure to decompose images into reflection components and illumination components. Among them, the illumination component reflects the slow illumination information of the overall face image. The reflection component reflects the authentic attributes of the face image. After the steps in Section 3.2.2, reconstructed images can be converted into light enhancement images and low illumination images. We paired face images in the training set with low-light face images, selected 100,000 pairs as the training set, and made pretraining of illumination enhancement and attenuation networks. The loss function *L*_*E*_ of the light enhancement network reached 0.091 and that of the light attenuation network hit 0.121. Both latent code generation network and face generation network adopted pretraining model. We also compared the method with StyleGAN1 [22] and StyleGAN2 [23] to demonstrate its state of the art. We set the hyperparameter *χ*_1_=1.0, *χ*_2_=0.5, *χ*_3_=0.3, and *χ*_4_=0.1.

### 4.2. Experimental Results and Analysis

The private face images *x*_*i*_′ generated by the method are shown in Table 2, and the deidentified face image ${\ddot{x}}_{i}$ was generated from a low-light face image *x*_*i*_ with the joint efforts of light enhancement network, latent code generation network, and face generation network. Although the facial characters had become visibly different from that of the original face image, they still maintained the basic appearance of the original face. Inputting deidentified face image into the light attenuation network, a low-light private face image was obtained. Attaching it to the original video or image, we found the styles of the two images are unified, which improved the user experience.

To test the privacy protection degree of the face privacy image *x*_*i*_′ generated by the method on face features, we applied the four pretrained face recognition networks in Table 1 respectively to recognize private face images. TPR and FPR were measured, as shown in Table 3.

It can be seen from Table 3 that if TPR declines while FPR rises, the proportion of correctly identified positive examples in total positive examples drops, and the percentage of negative examples predicted as positive one's increases. As such, private face image *x*_*i*_′ generated by this method can successfully mislead face recognition networks, thus protecting the privacy of users.

Low-light private face images generated by this method were compared with that by PGGAN [21], StyleGAN1 [15], and StyleGAN2 [16], as shown in Table 4. Because the method in this paper uses light enhancement and attenuation networks, the light of the face image generated by the method in this paper is more in line with the original image. It can be seen that the private face images generated by this method are more consistent with the original image in structural similarity than those generated by PGGAN, StyleGAN1, and StyleGAN2.

**Figure 5:**
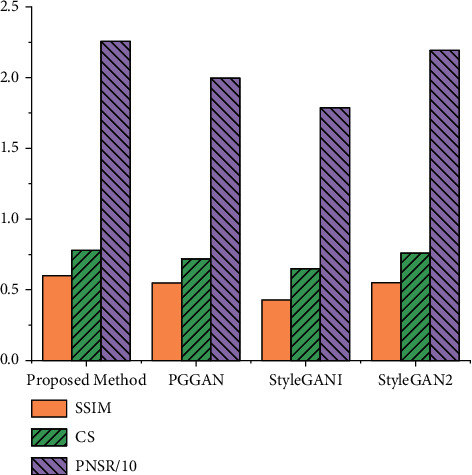
Comparisons of similarity between original face images and private face images generated by different methods.

The similarity comparison between private face images generated by this method and by PGGAN, StyleGAN1, and StyleGAN2 and the original face images is shown in Figure 5. In Figure 5, PSNR (Peak Signal to Noise Ratio) is the most common and widely used objective image evaluation index, which is the ratio of the energy of the peak signal to the average energy of the noise. SSIM (Structural Similarity) is a full-reference image quality evaluation index, which measures image similarity from three aspects: brightness, contrast, and structure. CS (Cosine similarity) calculates the angle between two vectors, which can be used to measure the direct similarity of images. The private face images generated by this method have higher SSIM, CS, and PSNR than those created by the other three methods. It indicates that the low-light face images generated by the proposed method are closer to the style of the original images and more real and natural, thus greatly improving the user experience.

**Figure 6:**
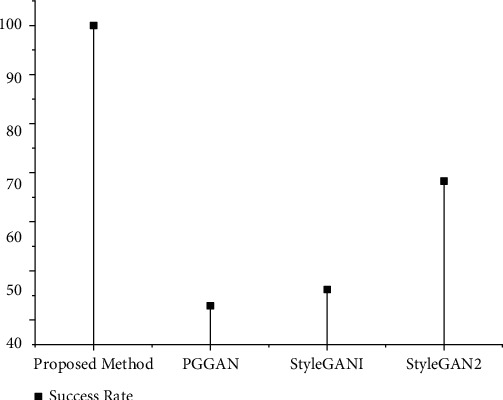
Comparisons of the success rate of low-light private face images generated by different methods.

The comparison of the success rate of the proposed method and PGGAN, StyleGAN1, and StyleGAN2 in generating low-light face deidentification images is shown in Figure 6. The success rate of the proposed method in generating low-light private face images is 100 percent, while that of PGGAN is 42.9%, StyleGAN1 is 46.2%, and StyleGAN2 is 68.3%. The front-facing face cropping network of PGGAN, StyleGAN1, and StyleGAN2 cannot completely detect the face area under low light, failing the generation of low-light images sometimes. Or the generated face images are completely black and cannot be recognized, resulting in generation failure. In our method, since the light enhancement network has enhanced the illumination of low-light photos, the front face region interception network is not affected by low light, and the success rate of detecting the face region is 100%. Moreover, under the effect of the light attenuation network, the style of the generated low-light face image is closer to the original face image.

## 5. Conclusion

This paper puts forward a low-light face image generation method based on deidentification. It overcomes the adverse effects of low light and generates face images of a low-light style, making private face images real and natural and thereby improving the user experience. Meanwhile, it reduces the accuracy of the face recognition network to protect the privacy of users. When IoT devices collect face images for internal storage, or IoT applications transmit face images through an external communication network, even if there is a storage data leakage or a man-in-the-middle attack, the method proposed in this article can effectively prevent the leakage of user privacy. The method in this paper can be applied to various application scenarios of face image collection, and it is an effective supplement to the existing face privacy image methods. In the future, we will do more research on lightweight models in private face image generation and optimize the operating speed to make its application more efficient in edge computing. In addition, there are deidentification methods in many special scenes, such as profile and occlusion, which need to be studied.

## Figures and Tables

**Table 1:** Accuracy and threshold of four face image recognition networks.

Face recognition model	Training accuracy	Test accuracy	TPR	FPR
VGG16	0.982	0.951	0.938	0.081
Resnet50	0.990	0.979	0.973	0.062
MobileNet V3	0.999	0.987	0.997	0.031
Senet50	0.975	0.960	0.917	0.172

**Table 2:** Private face images generated by the method.

Low-light face image	Deidentified face image	Private face image
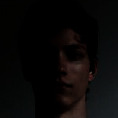	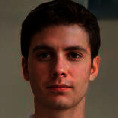	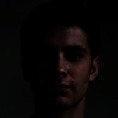
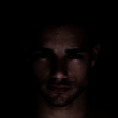	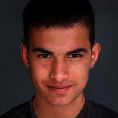	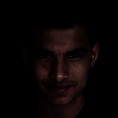
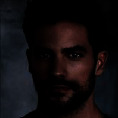	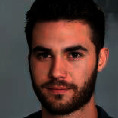	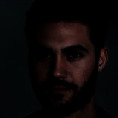
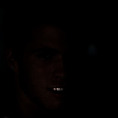	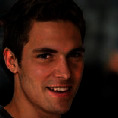	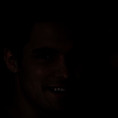
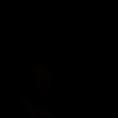	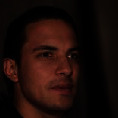	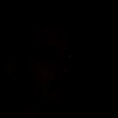
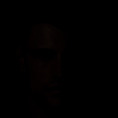	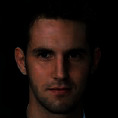	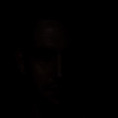

**Table 3:** TPR and FPR of four face recognition models to recognize private face images.

Face recognition model	TPR	FPR
VGG16	0.148	0.850
Resnet50	0.145	0.887
MobileNet V3	0.119	0.815
Senet50	0.103	0.854

**Table 4:** Comparisons of low-light private face images generated by different methods.

Method	Low-light face image	Private face image	Low-light face image	Private face image
The proposed method	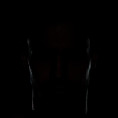	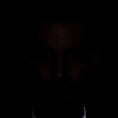	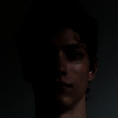	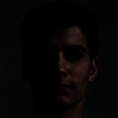
PGGAN	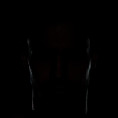	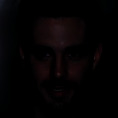	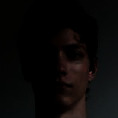	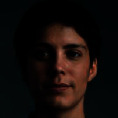
StyleGAN1	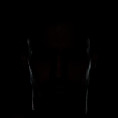	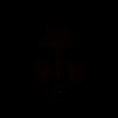	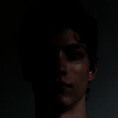	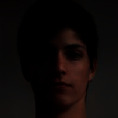
StyleGAN2	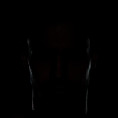	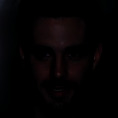	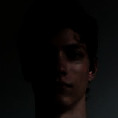	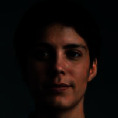

## Data Availability

The data used to support the findings of this study are available from the corresponding author upon request.
